# Propofol Versus Methohexital in Electroconvulsive Therapy: Impact on Treatment Efficacy and Adverse Effects. A Systematic Literature Review and Meta‐Analysis

**DOI:** 10.1111/aas.70083

**Published:** 2025-07-01

**Authors:** Saara H. Huoponen, Katrin Sisa, Tom Saari, Markku Taittonen, Ulla Ahlmén‐Laiho

**Affiliations:** ^1^ Department of Anesthesiology and Intensive Care University of Turku Turku Finland; ^2^ Perioperative Services, Intensive Care and Pain Medicine Turku University Hospital Turku Finland

**Keywords:** depression, electroconvulsive therapy, methohexital, propofol

## Abstract

**Background:**

Electroconvulsive therapy (ECT) is a widely used treatment for depression, but the choice of the anesthetic that is used for induction may affect both clinical outcomes and the occurrence of adverse effects (AEs). Propofol and methohexital are frequently used in Finland, yet their relative impact on treatment efficacy and AEs remains uncertain.

**Methods:**

We conducted a systematic literature review and meta‐analysis following Preferred Reporting Items for Systematic Reviews and Meta‐Analyses (PRISMA) guidelines. Electronic databases were searched up to January 21, 2025. Studies comparing propofol and methohexital in adult patients receiving ECT for depression and utilizing numeric scales for depression assessment were included. The primary outcome was the clinical treatment response, defined by the number of ECT sessions required to achieve remission. The secondary outcome was the variation of AEs associated with ECT between comparator groups.

We included eight studies in the final analysis with 194 patients in the propofol group and 198 patients in the methohexital group. Five of the studies were randomized controlled trials and three were retrospective cohort studies. Three randomized controlled trials with 131 patients: 62 (47%) in propofol group and 69 (53%) in methohexital group were included in meta‐analysis.

**Results:**

The number of ECT sessions required for recovery did not differ between groups. All studies demonstrated effective alleviation of depression through ECT, regardless of anesthetic choice. However, AEs were inconsistently reported, and a comprehensive overview of the topic was not possible.

**Conclusions:**

Low‐quality evidence suggests equal efficacy of propofol compared to methohexital with regard to clinical remission of depression after ECT.

**Systematic Review Registration:**

**Trial Registration:** PROSPERO; CRD42024520709.

**Editorial Comment:**

This systematic review and meta‐analysis presents the available but limited and low‐quality evidence in this study area, and supports an interpretation that propofol and methohexital have similar efficacy when facilitating electroconfulsive therapy as treatment for depression, to relieve depression symptoms.

## 1 Introduction

Anesthesia agents used for patients undergoing electroconvulsive therapy (ECT) have been a subject of research and discussion. Common anesthetics employed in ECT worldwide include thiopental, methohexital, ketamine, propofol, and etomidate. The properties of these agents vary, and while ketamine, for example, has demonstrated benefits in alleviating depression, it is also associated with a greater number of adverse effects (AEs) [1, 2].

In late 2022, a survey of Finnish neuromodulation units revealed two predominant strategies for anesthetic selection in ECT in Finland: most units (*n* = 14/78%) primarily used propofol, while others (*n* = 3/17%) favored methohexital [3]. These findings highlight a division in opinion regarding the optimal ECT anesthetic. The survey also reported significant variability in the incidence of ECT‐related AEs across units [4], raising the question of whether anesthetic choice influences AE occurrence.

Methohexital has been widely used as an ECT anesthetic since the 1960s [5], and many sources still declare it a gold standard [6]. However, some studies suggest that propofol may offer advantages, particularly in hemodynamic stability during ECT [7, 8]. Furthermore, propofol has shown to possess antiemetic properties in the management of surgical patients [9]. Considering that some ECT patients experience AEs such as nausea and vomiting, this aspect becomes particularly important [4, 10].

Concerns about propofol's suitability for ECT arose in the 1980s and 1990s [11, 12], as it was found to shorten EEG and EMG seizure duration. There is still no complete consensus on the effect of seizure duration on ECT treatment outcomes. Some studies suggest that seizure duration does not significantly affect treatment outcomes [13, 14], while others indicate that seizure duration is a predictor of treatment response, with short seizures being associated with worse outcomes [15].

In 2016, Fond et al. conducted a meta‐analysis [16] suggesting methohexital's superiority over propofol as an ECT anesthetic. However, this conclusion relied on only two RCTs after one was excluded for heterogeneity, and neither original study favored methohexital outright; both reported equal effectiveness in practice. Similarly, a 2017 systematic review and meta‐analysis by Lihua et al. combined data from four small RCTs and found no significant difference between methohexital and propofol in depression alleviation [17]. The authors noted that the included studies were of low quality, limiting the reliability of their findings.

Given the divided opinions among Finnish neuromodulation providers and the variability in reported ECT‐related AEs, we revisited the published research to conduct a new systematic review. Our goal was to identify whether recent or supplementary data could provide insights to support evidence‐based decisions regarding the choice of anesthetic for ECT. Since anesthetics other than propofol and methohexital were used infrequently in Finnish neuromodulation units, we decided to limit the comparison to these two anesthetics.

## 2 Methods

### 2.1 Registration

This systematic review, along with its protocol, follows the Preferred Reporting Items for Systematic Reviews and Meta‐Analyses (PRISMA) guidelines and has been officially registered in the International Prospective Register of Systematic Reviews (PROSPERO) under the registration number CRD42024520709.

### 2.2 Objective

This study aimed to compare propofol and methohexital as anesthetics for ECT in patients with depression. The primary outcome was the clinical treatment response. It was defined as the number of ECT sessions needed for remission and the reduction in numeric depression scores. The secondary outcome focused on comparing the variety, incidence, and severity of AEs between the propofol and methohexital groups.

### 2.3 Eligibility Criteria

All original studies meeting the eligibility criteria were considered, with no restrictions on language or publication date. Studies were deemed eligible for inclusion if they:

1. Compared propofol and methohexital as anesthetics for ECT.
2. Used validated numerical scales to assess depression outcomes.

Excluded materials included review articles, meta‐analyses, case reports, conference abstracts, letters, correspondences and nonpeer‐reviewed reports. Studies involving patients under 18 years old or those receiving ECT for nondepressive conditions were excluded. Trials with multiple comparator groups were not excluded, as long as they included at least propofol and methohexital groups.

### 2.4 Information Sources

We conducted systematic literature searches in PubMed/Medline, Embase, Cochrane Library, Scopus, PsycINFO, and Web of Science from inception to January 21, 2025. Reference lists of selected studies and systematic reviews were also screened to ensure comprehensive coverage.

### 2.5 Search Strategy

Search terms included “propofol,” “methohexital,” “electroconvulsive therapy (ECT),” and synonyms. Detailed search strategies, terms, and strings are provided in Appendix S1. Terms like “depression,” “adverse effects,” or specific depression scales were accounted for during data screening rather than the initial search. No filters were applied.

### 2.6 Selection Process

Search results were imported into Zotero, a reference management software. Zotero was used to identify duplicate records, after which they were removed. Two researchers (S.H.H. and K.S.) independently screened titles and abstracts, excluding articles that clearly did not meet inclusion criteria. Remaining articles underwent full‐text screening, which both researchers conducted independently. If there were discrepancies, they were resolved through discussion. The first researcher (S.H.H.) reviewed the reference lists of included studies and relevant systematic literature reviews for additional eligible studies.

### 2.7 Data Collection

A standardized template was developed for data extraction and the first researcher (S.H.H.) conducted the initial data extraction for all included articles.

Extracted data included:

1. Mean depression scores before and after ECT treatment series in both groups.
2. Mean number of ECT treatments needed to achieve remission.
3. Reported ECT‐related AEs, their incidence and severity.
4. Authors' conclusions.

Only depression scores measured before the start of the ECT course and the first score obtained after the final ECT session were included in the cases where multiple measurements were reported at different time points. We anticipated that studies would use different depression assessment scales to evaluate the outcome of ECT treatment. Eligible scales were:

1. MADRS (The Montgomery‐Åsberg Depression Rating Scale).
2. HDRS (The Hamilton Depression Rating Scale).
3. CGI (The Clinical Global Impression Scale).
4. BDI (The Beck Depression Inventory).

Please refer to Table 1 for more detailed information on the scales.

**TABLE 1:** Depression assessment scales overview.

Scale name	Description	Score range	Interpretation
Montgomery‐Åsberg depression rating scale (MADRS)	A 10‐item structured questionnaire that assesses the severity of depression. Each question can score from 0 to 6 points.	0–6 per question (total: 0–60)	Higher scores indicate greater severity of depression. **Reduction > 50% or a score of ≤ 10 indicates remission**
Hamilton depression rating scale (HDRS)	A 29‐item scale used to assess depression severity in patients.	0–4 per item (total: 0–116)	A higher score indicates more severe depression. **A Score of** ≤ **8 indicates remission**
Beck depression inventory (BDI)	A self‐administered questionnaire with 21 items, each allowing for a maximum of 3 points.	0–3 per item (total: 0–63)	Higher scores indicate more severe depression. **A Score of ≤ 10 indicates remission**
Clinical global impression scale (CGI)	Assesses the degree of mental illness or the progression of illness. It has 7 options: 1 (complete health) to 7 (most severe illness).	1–7	For illness progression: 1 (fully recovered), 7 (significant deterioration). Experienced clinicians use this scale. **A Score of** ≤ **2 indicates remission**

If a study utilized multiple depression scales, data from scales assessed by healthcare professionals (MADRS, HDRS, CGI) were prioritized over patient self‐reporting scales (BDI). No data conversion was applied to the depression scores.

### 2.8 Risk of Bias Assessment

Risk of bias was evaluated using the Mixed Methods Appraisal Tool (MMAT). It is a validated tool used to assess the quality of mixed‐methods research studies, which can combine, for example, both qualitative and quantitative approaches. It also includes specific criteria for evaluating various study designs, such as qualitative research, randomized controlled trials, nonrandomized studies, and retrospective studies. The MMAT uses a scoring system to help researchers systematically evaluate the methodological quality of studies, making it particularly useful in systematic reviews and evidence synthesis [18]. It allows assessing the risk of bias in different study designs. Two researchers (S.H.H. and K.S.) assessed the studies independently with discrepancies resolved by a third researcher (T.S.). No automated tools were used.

### 2.9 Effect Measures

For meta‐analysis, Hedge's g was calculated using reported means and standard deviations (SD). If standard errors (SEs) were reported instead, they were converted to SD. The study demographics, individual results from studies and incidence and severity of AEs were summarized in matrix tables with string and numerical variables.

### 2.10 Synthesis Methods

The study demographics, individual results from studies and incidence and severity of AEs were summarized in matrix tables with string and numerical variables. Findings were categorized by study design (RCTs and retrospective cohort studies). In meta‐analysis we used SPSS software to calculate and create a forest‐plot to report effect size. Other outcomes not included in meta‐analysis were presented narratively and supported by tables.

### 2.11 Reporting Bias and Heterogeneity Assessment

Alongside the forest plot, a funnel plot was created to assess heterogeneity and report small study/publication bias. If funnel plot asymmetry was detected, we planned to review trial and a characteristics to determine whether the asymmetry was due to publication bias or heterogeneity factors, such as methodological or clinical heterogeneity between studies. Heterogeneity was quantitatively assessed using the Cochran *Q* test, the *I* ^2^ statistic, and Tau^2^.

### 2.12 Certainty Assessment

The first researcher (S.H.H.) assessed the certainty of the evidence following the Grading of Recommendations Assessment, Development, and Evaluation system (GRADE). It is a validated system, used to assess the quality of evidence and strength of recommendations in healthcare [19]. GRADE was applied to the meta‐analysis outcome. The evidence was categorized as very low, low, moderate, or high quality. Evidence from RCTs is initially considered high quality. However, if there are factors that significantly reduce confidence in a study's results—such as serious risk of bias, inconsistency, indirectness, imprecision, or other issues like publication bias—each lowers the quality of evidence by one level: from high to moderate, low, or very low.

## 3 Results

### 3.1 Study Selection

**FIGURE 1:**
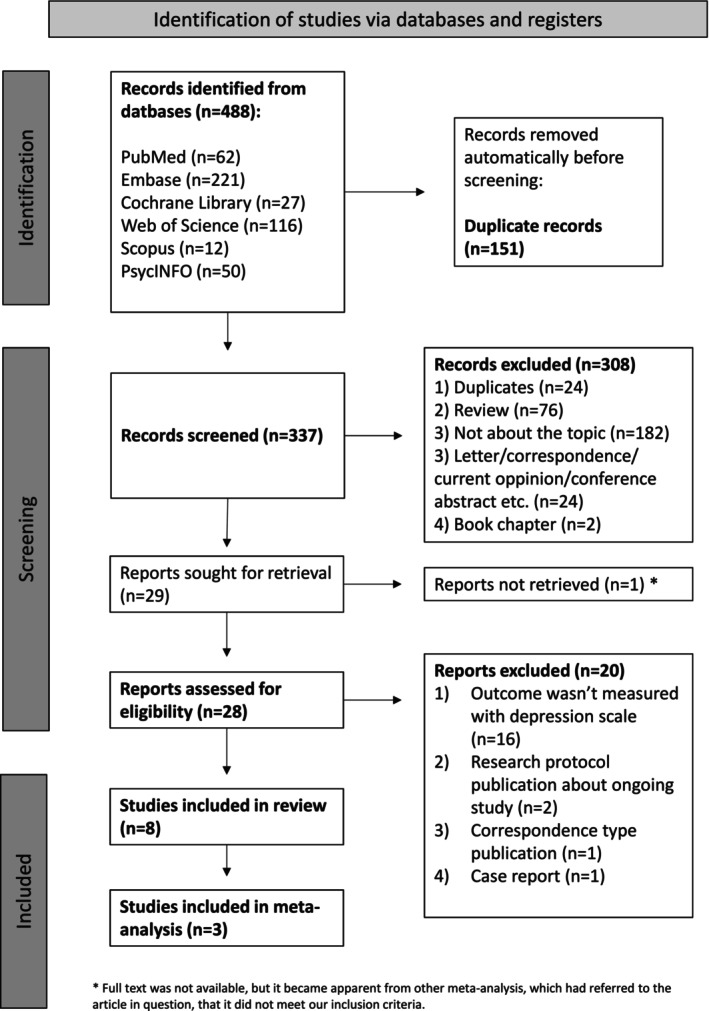
Preferred reporting items for systematic reviews and meta‐analysis (PRISMA) flow diagram.

Totally, 488 potential studies were identified through database searches. After removing 151 duplicates, we screened the remaining 337 records. Of these, 28 full‐text articles were reviewed, and eight met the inclusion criteria. Reference searches of the included studies and relevant systematic reviews did not yield additional eligible articles. The PRISMA flow diagram illustrating the study selection process is shown in Figure 1.

Additionally, 20 studies initially appeared eligible but were excluded after thorough content screening. Reasons for their exclusion are detailed in Appendix S2.

### 3.2 Study Characteristics and Risk of Bias

Of the eight eligible studies, five were randomized controlled studies [13, 20, 21, 22, 23] and three were retrospective cohort studies [24, 25, 26]. The MMAT was utilized to assess risk of bias for each of the included studies. Overall, seven studies [13, 20, 21, 22, 23, 24, 26] elicited concerns about bias, with three of them [21, 23, 24] eliciting major concerns. It should also be noted that although Vaidya's study [25] received 100% MMAT scores, the study design was retrospective, which may affect its reliability. A summary of risk of bias assessments is provided in Table 2.

**TABLE 2:** Mixed methods appraisal tool: Explanation and results from individual studies.

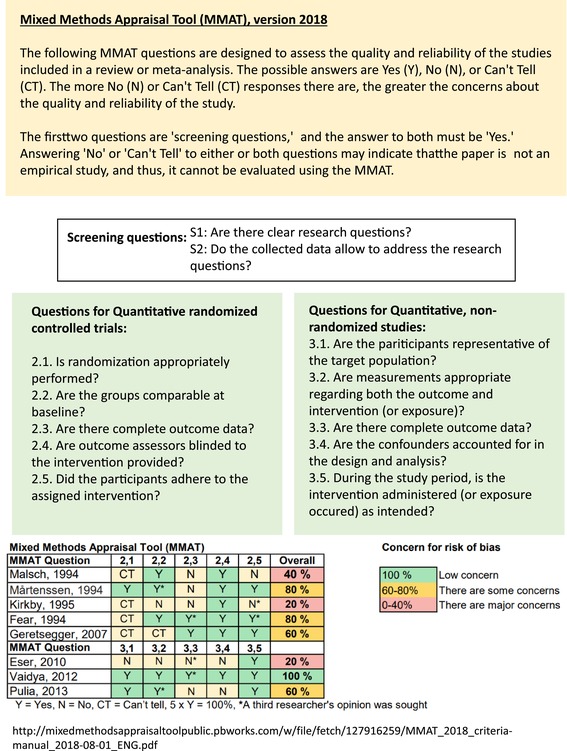

### 3.3 Results of Individual Studies and Synthesis

The literature review included a total of 194 patients in the propofol group and 198 patients in the methohexital group. Four studies [13, 21, 22, 23] used the HDRS scale while three [20, 25, 26] used MADRS scale to assess depression. Only Eser's study [24] used GCI scale. The results of all five RCTs [13, 20, 21, 22, 23] supported the likelihood that methohexital and propofol are equally suitable as ECT anesthetics. In retrospective studies, Eser [24] favored propofol, while Vaidya and Pulia [25, 26] supported methohexital. Across all studies, depression scores significantly decreased from baseline in both propofol and methohexital groups, indicating effective alleviation of depression. Further details of study characteristics and individual study results are summarized in Table 3.

**TABLE 3:** Summary of study characteristics, results, and authors' conclusions.

Author (year)	Depression scale	Electrode placement	Number of patients propofol vs methohexital	DS before ECT series, mean (SD) propofol vs methohexital	DS after ECT series, mean (SD) propofol vs methohexital	Mean (SD) nr of ECTs needed propofol vs methohexital	Authors conclusions
Randomized controlled trials							
Fear (1994)	HDRS	BL	9/11	20.9 (6.4) vs. 24.7 (6.7)	7.9 (9.2) vs. 11.5 (10.8)	6.6 (2.6) vs. 7 (3.7)	Despite reducing seizure duration by over 25%, propofol showed no difference on the HDRS and BDI, indicating it remains a viable induction agent for ECT.
Malsch (1994)	HDRS	RUL/BL	29/29	27.5 (4.69) vs. 30.7 (6.89)	4.4 (5.12) vs. 6.9 (9.21)	7.48 (1.45) vs. 7.76 (1.78)	The finding that propofol does not affect efficacy and patients recover from depression at a similar rate to methohexital may surprise those who link seizure duration with adequacy.
Mårtensson (1994)	MADRS	RUL	24/29 (21/24)^a^	34.7 (8.3) vs. 32.8 (7.3)	12.6 (9.9) vs. 15.7 (12.0)	10.4 (2.6) vs. 9.6 (3.0)	Both propofol and methohexital are effective ECT agents, and reduced seizure duration does not justify discontinuing the use of propofol.
Kirkby (1995)	HDRS	RUL	19/18 (16/16)^a^	19.63 (5.7) vs. 21.10 (4.8)	7.31 (5.44) vs. 8.56 (7.51)	6 vs. 6	If further research confirms the findings of this study then propofol may be a suitable agent for ECT.
Geretsegger (2007)	HDRS	UL	25/25	26.5 (7.1) vs. 31.3 (8.2)	9.8 (6.9) vs. 13.7 (11.5)	NR	Our results indicate that propofol, with comparable seizure quality and minimal blood pressure increase, is an effective anesthetic for ECT.
Retrospective cohort studies							
Eser (2010)	CGI	UL/BL	180/81^b^	NR	2.25 vs. 1.25	NR	Patients who underwent propofol anesthesia had significantly better outcomes than those with methohexital anesthesia.
Vaidya (2012)	MADRS	RUL/BL	48/48 (within subject design)	27.4 (8.9) vs. 29.8 (9.5)	−14.3 (9.1) vs. −15.6 (12.9)^c^	8.5 (4.5) vs. 7.8 (4.6)	For RUL placement, methohexital anesthesia requires fewer ECT treatments for depression than propofol.
Pulia (2013)	MADRS	RUL/BL	40/38	31.42 (*n* = 24) vs. 31.91 (*n* = 34)	14.96 (*n* = 24) vs. 16.38 (*n* = 34)	10.58 vs. 9.26	Switching from propofol to methohexital and adjusting the charge dose regimen in RUL ECT may enhance recovery

Abbreviations: BL = bilateral, CGI = clinical global impression, DS = depression score, HDRS = Hamilton depression rating scale, MADRS = Montgomery–Åsberg depression rating scale, NR = not reported, RUL = right unilateral, SD = standard deviation, UL = unilateral.

^a^ Number of patients after dropouts.

^b^ Number of treatments.

^c^ Mean reduction of DS from the baseline.

### 3.4 Meta‐Analysis

**FIGURE 2:**
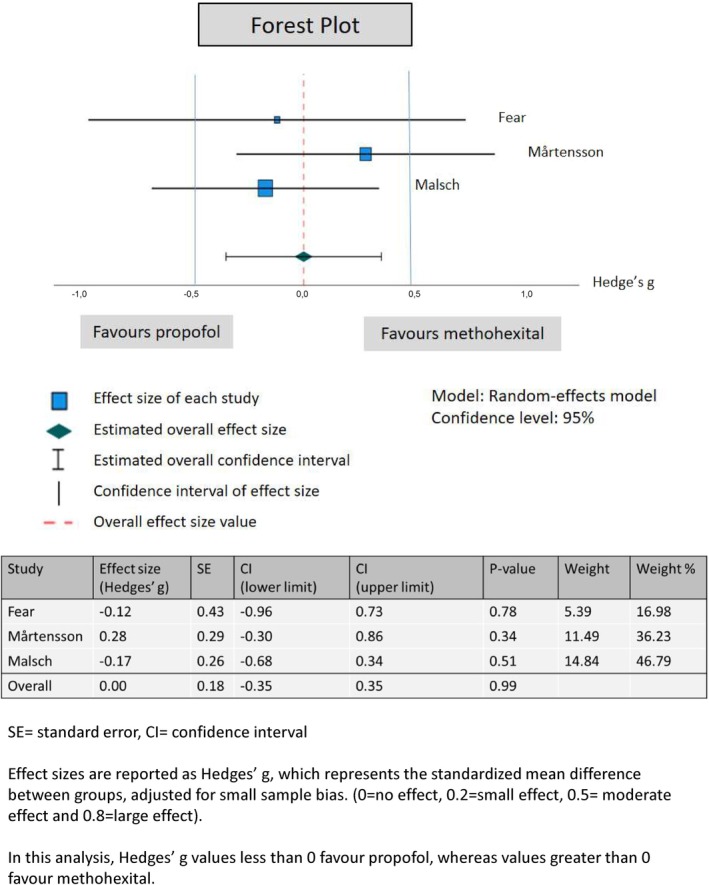
Meta‐analysis: the number of ECT sessions required for remission between methohexital and propofol groups.

The meta‐analysis of three RCTs [20, 21, 22], including 62 patients in the propofol group and 69 in the methohexital group, showed no significant difference in the number of ECT sessions required for remission between the two anesthetics. The overall effect size was negligible (Hedges' *g* = 0.00, SE = 0.18, *p* = 0.99), with a 95% confidence interval ranging from −0.35 to 0.35, indicating no advantage for either anesthetic (Figure 2).

Originally, we also planned to conduct a meta‐analysis comparing the reduction in depression scores between groups. However, our database search identified only one additional RCT by Mårtensson [20] compared to the previous meta‐analysis by Lihua [17], and as a result, a meta‐analysis about the topic was not conducted.

### 3.5 AEs

The studies included in this review provided limited information on the AEs associated with ECT treatment. Two RCTs [13, 20] and two retrospective cohort studies [24, 25] primarily addressed cognitive AEs. Neurocognitive outcomes varied: Mårtensson [20] and Vaidya [25] found no significant differences, while Geretsegger [13] noted a slight cognitive benefit with propofol. Eser [24] reported significantly less memory impairment with propofol. Studies by Geretsegger [13] found a more moderate blood pressure increase with propofol, and Eser [24] reported transient cardiovascular AEs in 11.5% of cases, with no significant group differences. However, common ECT‐related AEs such as headache, myalgia, nausea, or vomiting were not documented in any eligible study. Detailed information on AEs is summarized in Table 4.

**TABLE 4:** ECT‐related adverse effects: Conclusions from the studies.

Author	Evaluated adverse effects/used measurements	Conclusions
Fear 1994	NA	Propofol is better tolerated and provides greater hemodynamic stability, making it ideal for patients sensitive to changes.
Kirkby 1995	NA	Propofol may be especially suitable for patients where controlling hypertension during ECT is crucial, as it helps mitigate this complication.
Mårtensson 1994	MMSE and other cognitive test were done before, after fifth ECT and after entire ECT series to indicate possible variety in neuropsychological recovery	Neuropsychological tests showed no significant group differences. MMSE scores temporarily declined after the fifth ECT but normalized post‐treatment, with no lasting memory effects. Propofol may help reduce seizure‐related blood pressure increases, especially in high‐risk cardiovascular patients.
Geretsegger 2007	Boodpressure (BP) measurements. Several different tests (e.g., IQ, EOS, SST, STGI, REP) to assess cognitive function before and after ECT series.	Cognitive performance improved with propofol compared to methohexital, though only two tests showed significant differences. Blood pressure increases were also more moderate with propofol.
Malsch 1994	NA	NA
Eser 2010	Retrospective evaluation of cardiovascular and cognitive AEs	Transient cardiovascular AEs occurred in 11.5% of cases, with no significant group differences. Propofol caused less memory impairment than methohexital (*p* < 0.001), and was favored for its effective ECT outcomes and better tolerability.
Vaidya 2012	MMSE had been evaluated before and after ECT course to indicate cognitive impairment.	Mean MMSE scores before and after ECT were similar for methohexital and propofol anesthesia.
Pulia 2013	NA	NA

Abbreviations: AE = adverse effect, NA = not available.

### 3.6 Findings From Retrospective Cohort Studies

Retrospective cohort studies by Vaidya [25] and Pulia [26], conducted by the same research group and involving partially overlapping patient populations, reported that methohexital was associated with fewer ECT sessions required for remission compared to propofol when right unilateral (RUL) electrode placement was used. Pulia's study also noted shorter hospital stays in the methohexital group. Despite these findings, both anesthetics demonstrated similar efficacy in reducing depression scores.

Conversely, Eser's retrospective cohort study [24] reported significantly better outcomes (CGI scores) in the propofol group compared to methohexital.

### 3.7 Reporting Bias and Heterogeneity

To assess heterogeneity, potential small‐study effects and publication bias, we constructed an Egger's funnel plot. Visual inspection of the funnel plot showed symmetry and no indication of bias. Also the Cochran *Q* test, *I* ^2^, and Tau^2^ indicated no heterogeneity among the three included studies. Funnel plot is available in Appendix S3.

### 3.8 Certainty of Evidence (Meta‐Analysis)

According to the GRADE guidelines, the overall quality of evidence regarding the number of ECT sessions required for remission was rated as low Table 5.

**TABLE 5:** Grading of recommendation, assessment, development, and evaluation (GRADE).

Quality assessment regarding the topic “Number of ECTs needed for recovery”										
Number of studies	Studydesign	Risk of bias	Inconsistency	Indirectness	Impercicion	Other	Number of patients	Hedge's g	GRADE	Importance
3	RCT	Serious	Not serious	Serious	Not serious	None	63/69	0.00	**Low**	Important

## 4 Discussion

There are only a few studies comparing propofol and methohexital as anesthetics for ECT, with improvement in depression as an outcome. All of these studies were conducted over a decade ago. The methodology sections in older studies are relatively brief and lack detailed information. All of the studies also have small sample sizes, and it cannot be ruled out that chance may have played a role in influencing the results of these studies.

### 4.1 Comparison of Propofol and Methohexital

This review suggests that both propofol and methohexital are effective anesthetics for ECT. Depression scores improved similarly in both groups, and the number of ECT sessions required did not differ, indicating that anesthetic choice has no significant impact on treatment efficacy. The observed improvement appears to result from ECT itself rather than the anesthetic used. Furthermore, our meta‐analysis found no difference in the average number of ECT sessions needed for remission between the two anesthetics.

While overall effectiveness was similar, retrospective studies by Vaidya [25] and Pulia [26] identified scenarios in which methohexital may offer relative advantages. In the context of RUL electrode placement, methohexital was associated with fewer ECT sessions required for remission compared to propofol. However, the findings should be interpreted within the context of methodological constraints. They represent hypotheses that require confirmation in future high‐quality randomized controlled trials.

Conversely, Eser [24] reported better outcomes with propofol, but these findings are less robust due to methodological limitations and needs to be interpreted with caution. While the propofol group in Eser's study consisted primarily of patients with depression the methohexital group included also patients with schizophrenia. Additionally, Eser's data reflected the total number of ECT treatments sessions, rather than the number of patients in each comparator group, further limiting interpretability.

### 4.2 AEs

Considering the potential AEs and their frequency is crucial when evaluating the superiority of one anesthetic over another. AEs such as nausea, headache, myalgia, confusion, and cognitive impairment can affect patients' compliance with ECT treatment. Four studies [13, 20, 22, 23] suggested that propofol might be more suitable for patients with cardiovascular issues, such as high blood pressure, due to its ability to reduce post‐treatment hypertension. However, the studies included in this review provided limited information on ECT‐related AEs, preventing any definitive conclusions about the relative superiority of one anesthetic over the other in terms of AEs.

### 4.3 Clinical Implications

The absence of clear evidence favoring one anesthetic over the other is, in some ways, reassuring. Given that both propofol and methohexital are associated with significant and comparable alleviation of depression, differing clinical preferences for ECT anesthetics are unlikely to substantially affect patient outcomes.

The low quality of randomized trials investigating the effects of propofol and methohexital on ECT treatment outcomes and AEs underscores the need for further research.

## 5 Limitations

There were differences in study designs, depression scales used, patient characteristics, and the number of reported AEs, all of which limit the ability to synthesize the findings. The sample sizes in RCTs were small, ranging from 9 to 29 patients. Additionally, Eser [24] did not report the number of patients in the control groups but instead provided data on the total number of ECT treatment sessions.

Retrospective cohort studies were excluded from the meta‐analysis due to the inherent biases associated with their studydesign. However, despite these limitations, these studies were included in the qualitative synthesis to offer a more comprehensive overview, given the limited number of recent studies on this topic.

While the research protocol registered in PROSPERO aimed to examine ECT‐related AEs in propofol and methohexital groups, the eligible studies provided only limited information on this topic, making a quantitative analysis impossible.

## 6 Conclusions

Based on current studies, low‐quality evidence suggests that there is no significant difference between methohexital and propofol in terms of depression improvement or the number of ECT sessions required for remission. The lack of robust evidence highlights the need for well‐designed RCTs to provide clearer insights.

## Author Contributions

**Saara H. Huoponen:** conceptualisation, methodology, data collection, formal analysis, writing and editing of the original draft. **Katrin Sisa:** conceptualisation, methodology, formal analysis, editing of the original draft. **Tom Saari:** methodology, formal analysis, review of the original draft. **Markku Taittonen:** supervision, review and editing of the original draft. **Ulla Ahlmén‐Laiho:** supervision, conceptualisation, editing and review of the original draft.

## Conflicts of Interest

The authors declare no conflicts of interest.

## Supporting information


**Appendix S1.** Databases and search strategy.

**Appendix S2.** Studies that were finally excluded.

**Appendix S3.** Funnel plot to assess publication bias and small‐study effects.

## Data Availability

The data supporting the findings of this study are openly available from the databases cited throughout the article and included in the supporting information:appendices. Specific datasets and datasheets used in the analysis are available from the corresponding author upon reasonable request.
