# Supplementary material for: Propofol Versus Methohexital in Electroconvulsive Therapy: Impact on Treatment Efficacy and Adverse Effects. A Systematic Literature Review and Meta‐Analysis
Source: Acta Anaesthesiol Scand. 2025 Jul 1;69(7):e70083. doi: 10.1111/aas.70083 (PMC12214048; doi:10.1111/aas.70083)
Supplement: Supplementary file 1 — Appendix S1. Databases and search strategy. [file AAS-69-0-s002.docx]

**Appendix 1. Databases and search strategy.**

(searches run 21.1.2025)

**PubMed:**

((methohexital* OR methohexitone OR "Methohexital"[Mesh]) AND (((((2,6-diisopropylphenol*) OR (propofol*) OR (2,6-diisopropyl-4-(3-(3-methyl-3H-diazirin-3-yl)propyl)phenol [Supplementary Concept]) OR (Propofol[Mesh]))) ) OR (propofol[MeSH Terms]))) AND ((((((electroconvulsive therap*) OR (ECT)) OR (convulsive therap*)) OR (electroconvulsive shock[MeSH Terms])) OR (electroconvulsive therapy[MeSH Terms])) OR (shock, electroconvulsive[MeSH Terms]))

("methohexital*"[All Fields] OR ("Methohexital"[MeSH Terms] OR "Methohexital"[All Fields] OR "methohexitone"[All Fields]) OR "Methohexital"[MeSH Terms]) AND ("2 6 diisopropylphenol*"[All Fields] OR "propofol*"[All Fields] OR (("2 6 diisopropyl 4"[All Fields] AND (("3"[All Fields] AND "3-methyl-3H-diazirin-3-yl"[All Fields]) AND ("propyl"[All Fields] OR "propylated"[All Fields] OR "propylation"[All Fields] OR "propylic"[All Fields]))) AND "phenol"[Supplementary Concept]) OR "propofol"[MeSH Terms] OR "propofol"[MeSH Terms]) AND ((("electroconvulsant"[All Fields] OR "electroconvulsion"[All Fields] OR "electroconvulsions"[All Fields] OR "electroconvulsive"[All Fields]) AND "therap*"[All Fields]) OR "ECT"[All Fields] OR (("convulsants"[Pharmacological Action] OR "convulsants"[MeSH Terms] OR "convulsants"[All Fields] OR "convulsant"[All Fields] OR "convulse"[All Fields] OR "convulsed"[All Fields] OR "convulsing"[All Fields] OR "convulsive"[All Fields] OR "convulsives"[All Fields] OR "seizures"[MeSH Terms] OR "seizures"[All Fields] OR "convulsion"[All Fields] OR "convulsions"[All Fields]) AND "therap*"[All Fields]) OR "electroshock"[MeSH Terms] OR "electroconvulsive therapy"[MeSH Terms] OR "electroshock"[MeSH Terms])

62 records

**Embase:**

('electroconvulsive therapy'/exp OR 'electroconvulsive therapy') AND propofol AND methohexital

221 records

**COCHRANE LIBRARY:**

electroconvulsive NEXT therap* OR ECT in All Text AND (propofol* OR "2,6-diisopropylphenol") AND (methohexital OR methohexitone) in Title Abstract Keyword - (Word variations have been searched)

27 records

**Web of Science:**

((ALL=("electroconvulsive therapy" OR ECT OR "Electroconvulsive Therapy"[Mesh])) AND (ALL=((2,6-diisopropylphenol OR propofol OR "2,6-diisopropyl-4-(3-(3-methyl-3H-diazirin-3-yl)propyl)phenol" [Supplementary Concept] OR "Propofol"[Mesh])) AND ALL=(methohexital OR methohexitone OR "Methohexital"[Mesh])))

116 records

**SCOPUS:**

("electroconvulsive therap*" OR ect) AND (( propofol* OR 2,6-diisopropylphenol* ) AND ( methohexital OR methohexitone ))

12 records

**PsycINFO**

( "electroconvulsive therap*" OR ect OR "Electroconvulsive Therapy"[Mesh] ) AND ( methohexital OR methohexitone OR "Methohexital"[Mesh] ) AND ( propofol OR (2,6-diisopropylphenol) OR "Propofol"[Mesh] OR "2,6-diisopropyl-4-(3-(3-methyl-3H-diazirin-3-yl)propyl)phenol" [Supplementary Concept] )

50 records
