# Supplementary material for: Propofol Versus Methohexital in Electroconvulsive Therapy: Impact on Treatment Efficacy and Adverse Effects. A Systematic Literature Review and Meta‐Analysis
Source: Acta Anaesthesiol Scand. 2025 Jul 1;69(7):e70083. doi: 10.1111/aas.70083 (PMC12214048; doi:10.1111/aas.70083)
Supplement: Supplementary file 2 — Appendix S2. Studies that were finally excluded. [file AAS-69-0-s001.docx]

**Reports excluded from the analysis**:

1. Nguyen, T. T., A. K. Chhibber, S. J. Lustik, J. W. Kolano, P. J. Dillon, ja L. B. Guttmacher. ”Effect of Methohexitone and Propofol with or without Alfentanil on Seizure Duration and Recovery in Electroconvulsive Therapy.” *British Journal of Anaesthesia* 79, nro 6: 801–3.<https://doi.org/10.1093/bja/79.6.801>.

**No depression score was used**

1. Bailine, Samuel H., Georgios Petrides, Martin Doft, ja Gene Lui. ”Indications for the Use of Propofol in Electroconvulsive Therapy.” *The Journal of ECT* 19, 2003:129–32.<https://doi.org/10.1097/00124509-200309000-00002>.

**Propofol group had long seizures before switching anesthetic, no depression scores.**

1. Scott, Allan, ja Harold Boddy. ”Induction agents in electroconvulsive therapy: A comparison of methohexitone and propofol”. *Psychiatric Bulletin* 26, nro 12, 2002: 455–57.<https://doi.org/10.1192/pb.26.12.455>.

**No depression score was used**

1. Dwyer, R., W. McCaughey, J. Lavery, G. McCarthy, ja J.W. Dundee. ”Comparison of Propofol and Methohexitone as Anaesthetic Agents for Electroconvulsive Therapy”. *Anaesthesia* 43, nro 6, 1988: 459–62.<https://doi.org/10.1111/j.1365-2044.1988.tb06631.x>. **No depression score was used**
2. Lim, S. K., W. L. Lim, ja E. O. Elegbe. ”Comparison of Propofol and Methohexitone as an Induction Agent in Anaesthesia for Electroconvulsive Therapy.” *West African Journal of Medicine* 15, nro 4, 1996: 186–89.

**No depression score was used**

1. Rampton, A. J., R. M. Griffin, C. S. Stuart, J. J. Durcan, N. C. Huddy, ja M. A. Abbott. ”Comparison of Methohexital and Propofol for Electroconvulsive Therapy: Effects on Hemodynamic Responses and Seizure Duration.” *Anesthesiology* 70, nro 3, 1989: 412–17.<https://doi.org/10.1097/00000542-198903000-00008>.

**No depression score was used**

1. Swaim, Jennifer C., Maher Mansour, Salina M. Wydo, ja Jeffrey L Moore. ”A Retrospective Comparison of Anesthetic Agents in Electroconvulsive Therapy”. *The Journal of ECT* 22, nro 4, 2006: 243–46.<https://doi.org/10.1097/01.yct.0000244238.17791.a4>.

**No depression score was used**

1. Fear, C. F., C. S. Littlejohns, ja E. C. Rouse. ”Propofol and ECT.” *The British Journal of Psychiatry : The Journal of Mental Science* 162, 1993: 421–22.<https://doi.org/10.1192/bjp.162.3.421b>.

**Preliminary results, correspondence type publication**

1. Shteinlukht, T.P., U. Berth, ja R. Badr. ”Propofol and Methohexital as Anesthetic Agents for ECT in Older Adults”. *American Journal of Geriatric Psychiatry* 17, 2009: A92–93.<https://doi.org/10.1097/01.JGP.0000346964.46544.ec>.

**No depression score was used**

1. Geretsegger, Christian, Erika Rochowanski, Christopher Kartnig, ja Axel F. Unterrainer. ”Propofol and methohexital as anesthetic agents for electroconvulsive therapy (ECT): A comparison of seizure-quality measures and vital signs”. *The Journal of ECT* 14, 1998: 28–35.<https://doi.org/10.1097/00124509-199803000-00005>.

**No depression score was used**

1. Rouse, E.C. ”Propofol for Electroconvulsive Therapy. A Comparison with Methohexitone. Preliminary Report”. *Anaesthesia* 43, nro SUPPL. 1988: 61–64.

**No depression score was used**

1. Simpson, K.H., P.J. Halsall, C.M.E. Carr, ja K.G. Stewart. ”Propofol Reduces Seizure Duration in Patients Having Anaesthesia for Electroconvulsive Therapy”. *British Journal of Anaesthesia* 61, nro 3 1988: 343–44.

**No depression score was used**

1. Avramov, M.N., M.M. Husain, ja P.F. White. ”The Comparative Effects of Methohexital, Propofol, and Etomidate for Electroconvulsive Therapy”. *Anesthesia and Analgesia* 81, nro 3 1995: 596–602.<https://doi.org/10.1097/00000539-199509000-00031>.

**No depression score was used**

1. Luccarelli, J., T.H. McCoy, R.J. Horvath, S.J. Seiner, ja M.E. Henry. ”The Effects of Anesthetic Change on Electrographic Seizure Duration during Electroconvulsive Therapy”. *Brain Stimulation* 14, nro 5, 2021: 1084–86.<https://doi.org/10.1016/j.brs.2021.07.007>.

**No clear comparator groups (propofol vs. methohexital)**

1. ”Comparison of Esketamine/Propofol and Methohexital Anesthesia for ECT | Cochrane Library”. opened 12. March 2024.<https://www.cochranelibrary.com/central/doi/10.1002/central/CN-02508515/full>.

**Research protocol publication, ongoing study**

1. EUCTR2021-003676-13-AT. ”Comparison of Anesthesia with Methohexital to Anesthesia with a Mixture of Propofol and Ketamin for Electroconvulsive Therapy”. *Https://Trialsearch.Who.Int/Trial2.Aspx?TrialID=EUCTR2021-003676-13-AT*, Aug 31, 2022.<https://doi.org/10.1002/central/CN-02429498>.

**Research protocol publication, ongoing study**

1. Fredman, B., J. d’Etienne, I. Smith, M.M. Husain, ja P.F. White. ”Anesthesia for Electroconvulsive Therapy: Effects of Propofol and Methohexital on Seizure Activity and Recovery”. *Anesthesia and Analgesia* 79, nro 1, 1994: 75–79.

**Clinical outcome was not measured, only seizure duration.**

1. Matters, R. M., W. G. Beckett, K. C. Kirkby, ja T. E. King. ”Recovery after Electroconvulsive Therapy: Comparison of Propofol with Methohexitone Anaesthesia.” *British Journal of Anaesthesia* 75, nro 3 1995: 297–300.<https://doi.org/10.1093/bja/75.3.297>.

**There was a statement that HDRS had been used, but there were no numeric results available.**

1. Fredman, B, Husain MM and White PF.”Anesthesia for Electroconvulsive Therapy –Use of Propofol Revisited” European Journal of Anaestehesiology 11, nro 5,1994: 423–25.

**Case report type publication, only three patients were included.**

1. Hussein, Ali, ja E. Salib. ”Anaesthesia in ECT”. *International Journal of Psychiatry in Clinical Practice* 2, nro 4, 1998: 283–85.<https://doi.org/10.3109/13651509809115375>.

**Numerical depression score was not used**
