# Supplementary figures and images for: Propofol Versus Methohexital in Electroconvulsive Therapy: Impact on Treatment Efficacy and Adverse Effects. A Systematic Literature Review and Meta‐Analysis
Source: Acta Anaesthesiol Scand. 2025 Jul 1;69(7):e70083. doi: 10.1111/aas.70083 (PMC12214048; doi:10.1111/aas.70083)

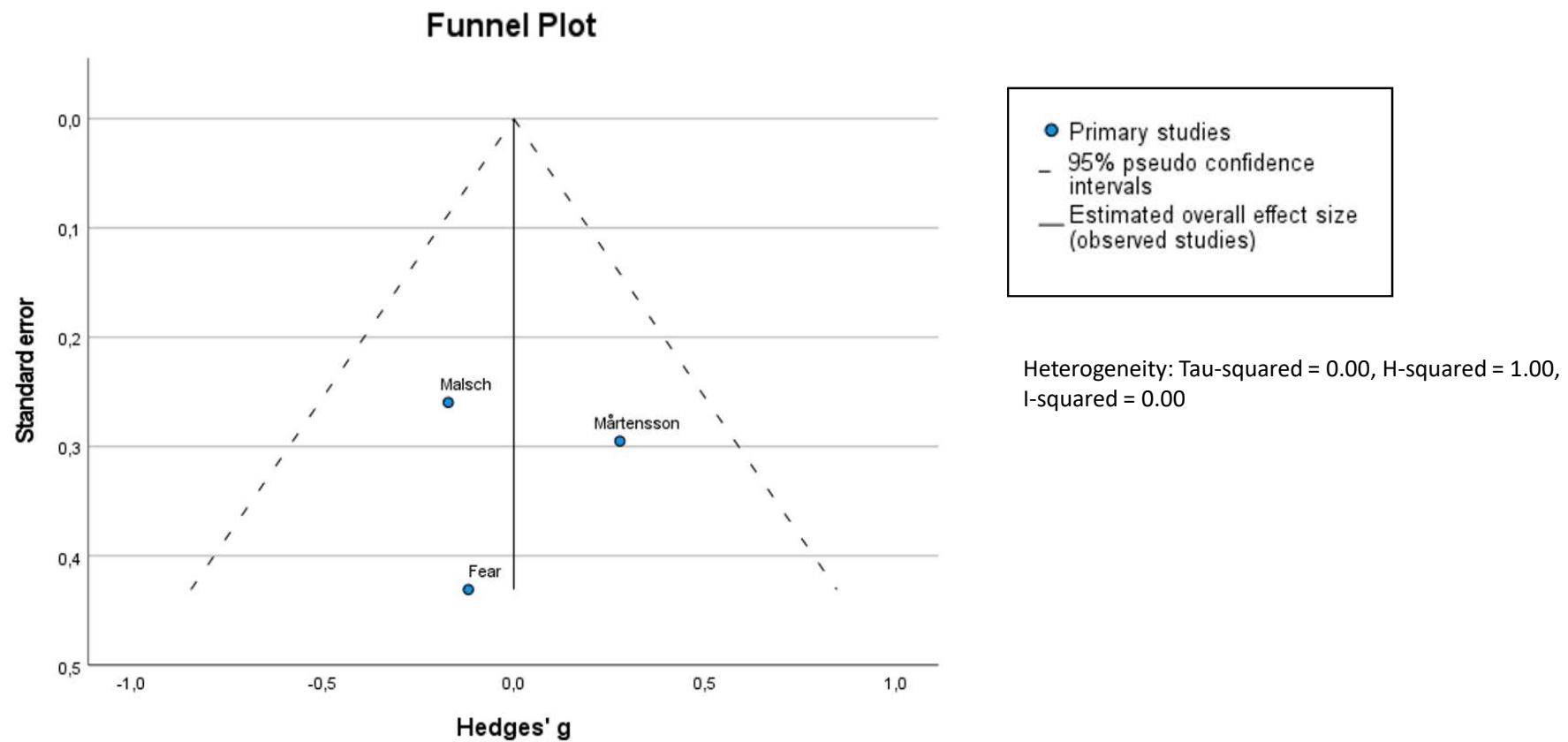

**Appendix 3.** Funnel plot to assess publication bias and small-study effects

Supplement: Supplementary file 3 — Appendix S3. Funnel plot to assess publication bias and small‐study effects. [file AAS-69-0-s003.pdf]
